# Comparing neoadjuvant long-course chemoradiotherapy with short-course radiotherapy in rectal cancer

**DOI:** 10.1186/s12876-021-01851-0

**Published:** 2021-07-07

**Authors:** Jian Wang, Yiwen Long, Kun Liu, Qian Pei, Hong Zhu

**Affiliations:** 1 Department of Oncology, Xiangya Hospital, Central South University, Changsha, 410008 Hunan China; 2 Department of Gastrointestinal Surgery, Xiangya Hospital, Central South University, Changsha, 410008 Hunan China

**Keywords:** Rectal cancer, Neoadjuvant therapy, Radiotherapy

## Abstract

**Background:**

The purpose of this study was to compare short-course radiotherapy (SC) or neoadjuvant long-course chemoradiotherapy (LC) treatment for locally advanced rectal cancer patients.

**Methods:**

Patients with a diagnosis of locally advanced rectal cancer (LARC) who had undergone neoadjuvant radiotherapy before surgery between 2013 and 2018 at the medical center in China were included in this study. All patients’ MRI confirmed T2N+M0 or T3-4N0-3M0 clinical stages. Patients in the SC group received pelvic radiotherapy with a dose of 5 × 5 Gy (with or without chemotherapy at any time), followed by immediate or delayed surgery. Patients in the LC group received a dose of 50–50.4 Gy in 25–28 fractions, concomitantly with FOLFOX or capecitabine-based chemotherapy, followed by surgery 4–6 weeks later. All clinical data were retrospectively collected, and long-term follow-up was completed and recorded at the same time.

**Results:**

A total of 170 were eligible to participate in this study, 32 patients in the SC group, and 138 in the LC group. The median follow-up time of living patients was 39 months. The disease-free survival (DFS) and overall survival (OS) rates in the SC group and LC group at 3 years, were, 84.9% versus 72.4% (*P* = 0.273) and 96.2% versus 87.2% (*P* = 0.510), respectively. The complete pathological response (pCR) rates in the SC group and LC group were, 25% versus 18.1% (the difference was not statistically significant, *P* = 0.375), respectively. However, the SC group had better node(N) downstaging compared to the LC group (*P* = 0.011).

**Conclusions:**

There were no differences observed in DFS and OS between short-course radiotherapy and long-course chemoradiation, and both can be used as treatment options for patients with locally advanced rectal cancer.

## Background

Colorectal cancer is one of the most common malignant tumors, accounting for 1/10 of all cancer cases and deaths [1]. In China, the incidence rate of colorectal cancer is high, and it is ranked as one of the five main causes of cancer-related deaths [2]. Most of the patients are diagnosed at the locally advanced middle and late stages, which results in the poor prognosis of the patients.

Over the past few decades, as the management of rectal cancer has significantly evolved, and neoadjuvant therapy including radiotherapy and chemotherapy has become an indispensable part of the treatment. CAO/ARO/AIO-94 III phase trial [3] compared preoperative and postoperative radiotherapy and chemotherapy in patients with locally advanced rectal cancer. The recurrence rate and acute and chronic toxicity of the preoperative chemoradiotherapy group was found to have significantly decreased, and at 5 years, the cumulative local recurrence rate was reduced, establishing the status of preoperative radiotherapy and chemotherapy in locally advanced rectal cancer. Nowadays, preoperative radiotherapy followed by total mesorectal excision is the standard treatment for locally advanced rectal cancer. There are two standard preoperative therapy options, including short-course radiotherapy (5 × 5 Gy) with immediate or delayed surgery, and long course chemoradiotherapy (45–50 Gy) with concurrent chemotherapy and surgical treatment after 4–8 weeks. Short course radiotherapy is the most preferred option in Europe [4], while long course chemoradiotherapy is majorly supported in the United States [5]. Short-course has the advantages of reduced cost and improved patient convenience as treatment is completed within a shorter time, while long-term chemoradiotherapy is closely related to higher sphincter preservation and lower surgical morbidity. However, some prospective studies have demonstrated that there is no difference in long-term oncological outcomes [6, 7]. The current Chinese guidelines recommend long-term simultaneous radiotherapy and chemotherapy as grade I for preoperative treatment of middle and low rectal cancer in cT3/T4N + , while short-term radiotherapy is recommended as grade II, and needs to be discussed in many disciplines before implementation [8].

Although both short-course preoperative radiotherapy and long-course preoperative chemoradiotherapy have been practiced in parallel in the past few decades, it is not clear which is the best neoadjuvant therapy for locally advanced rectal cancer. In this retrospective study, we compared short-course radiotherapy with long-course chemoradiotherapy, to determine the most beneficial therapy for locally advanced rectal cancer. In this study, the results were reported after a minimum follow-up period of 3 years, and a comparison of disease-free survival, overall survival, complete pathological response rate, and tumor and lymph node descending stage was also included.

## Methods

### Patient selection

In this retrospective single-institution cohort study with long-term follow-up, patients with a diagnosis of locally advanced rectal cancer (LARC) who had undergone neoadjuvant radiotherapy before surgery between 2013 and 2018 at the medical center in China were included in this study. The eligibility criteria were as follows: histologically confirmed rectal carcinoma, lower border within 10 cm from the anal verge, magnetic resonance imaging (MRI) confirmed clinical T2/N+ or T3-4/any N. The exclusion criteria included evidence of distant metastases, recurrent rectal cancer, unknown clinical or pathological T and N category or missing follow-up data.

All relevant data, including patient’s demographic data, clinical stage, and characteristics, as well as neoadjuvant therapy and surgical methods, were retrieved from the hospital’s patient records and recorded in detail.

### Treatment and follow-up

Eligible patients received either short-course radiotherapy (SC) or long-course chemoradiotherapy (LC) as neoadjuvant therapy. SC was defined as five fraction radiotherapy to a total dose of 25 Gy over five days, (with or without chemotherapy as part of their treatment course), followed by immediate surgery (within 4 weeks) or delayed surgery (more than 4 weeks).

LC comprised a total of 50–50.4 Gy in 25–28 fractions administered 5 days per week for the duration of radiation concomitantly with FOLFOX or capecitabine-based chemotherapy, followed by surgery 4–6 weeks later.

The radiation clinical target volume (CTV) included the primary rectal cancer, perirectal and internal iliac nodes, mesorectum, pelvic sidewalls, and presacral space with the upper border at the sacral promontory. Intensity-modulated radiotherapy (IMRT) using 6–18 MV photons was used, with daily image guidance.

Follow-up data were collected based on clinical examination or by telephone every 3 to 6 months after discharge, and dates of death were verified using data obtained from the census registry office.

### TNM staging

Staging of rectal cancer was performed according to the Union for International Cancer Control/American Joint Committee of Cancer (UICC/AJCC) 8.0. The clinical stage of the neoplasm was assessed in preoperative examinations performed before radiotherapy: endorectal ultrasound, colonoscopy, abdominal ultrasound, chest x-ray, and pelvic MRI (Done by both observers with inter-observer agreement). Pathological TNM (ypTNM) was determined by a histologist after the assessment of the specimen. T and N downstaging was recorded when the pathological stage was lower than the clinical stage before neoadjuvant treatment. Complete pathological response (pCR) was defined as the absence of a residual tumor at the time of the histological examination of the resected specimen [9].

### Statistical methods

The median and interquartile range (IQR) were used to express the continuous variables, and Kolmogorov–Smirnov test was used to detect the normal distribution, and t-test was used for those that conformed to the normal distribution, otherwise Mann–Whitney *U* test was used. The *χ*^2^ test or Fisher exact test was used to analyze the categorical variables. Survival data were analyzed using the Kaplan–Meier method and the log-rank test was used to detect differences between groups. All tests were two-sided and *P* < 0.05 was considered statistically significant. All statistical analyses were performed using the SPSS software (SPSS, version 24.0; SPSS Inc, Chicago, IL, USA).

Disease-free survival (DFS) was defined as the time from the date of operation to the time of confirmed local recurrence, distant metastases, or death due to disease or treatment. At the last follow-up, patients who were alive and disease-free (or died of non-rectal cancer causes, with no evidence of disease) were censored. Overall survival (OS) was defined as the time from the beginning of radiotherapy to death from any cause, with survivors being censored at the last follow-up time.

## Results

### Demographic data

Between 2013 and 2018, 170 patients were enrolled (SC, 32; LC, 138). Table 1. presents the patients’ characteristics between the two groups (age, sex, clinical staging, tumor localization, the level of CEA, mode, and type of operation). There were slightly more male patients (55.9% males), and most patients had a low tumor (63.5%, within 5 cm from the anal verge). The majority of the patients underwent laparoscopic surgery (53.5%) and Dixon surgery (62.9%), and 70.6% of the patients were confirmed to have stage T3, while 87.1% had lymph node-positive disease.

**Table 1:** Patients’ characteristics at different radiotherapy duration

Clinical variable	Radiotherapy duration		χ^2^	P value
	SC (%)	LC (%)		
Age Median (IQR), years	59 (49–65)	53 (45–60)	–	0.01*
*Sex*				
Male Female	18 (56.25) 14 (43.75)	87 (63.04) 51 (36.96)	0.51	0.48**
*Distance to anus*				
≤ 5 cm > 5 cm	23 (71.88) 9 (28.12)	85 (61.59) 53 (38.41)	1.19	0.28**
*Mode of operation*				
Open abdomen Endoscopic	6 (18.75) 26 (81.25)	73 (52.90) 65 (47.10)	12.18	< 0.01**
*Type of operation*				
Dixon Hartmann Miles	20 (62.50) 0 (0.00) 12 (37.50)	87 (63.04) 4(2.90) 47(34.06)	–	0.93***
*Neoadjuvant chemotherapy*				
No Yes	5 (15.63) 27 (84.37)	36 (26.09) 102 (73.91)	1.55	0.21**
CEA Median (IQR), ng/mL	2.74 (1.55–5.39)	3.95 (1.81–15.27)	–	0.11*
*Clinical tumor stage*				
cT2 cT3 cT4	4 (12.50) 23 (71.88) 5 (15.62)	4 (2.90) 97 (70.29) 37 (26.81)	5.42	0.08**
*Lymph node status*				
Negative Positive	2 (6.25) 30 (93.75)	20 (14.49) 118 (85.51)	0.92	0.34**

CEA, carcinoembryonic antigen; SC, short-course radiotherapy; LC, Long-course chemoradiotherapy; IQR, interquartile range

*: Mann–Whitney *U* test, **: *χ*^2^ test, ***: Fisher exact test

### Disease-free survival rate and overall survival

**Fig. 1:**
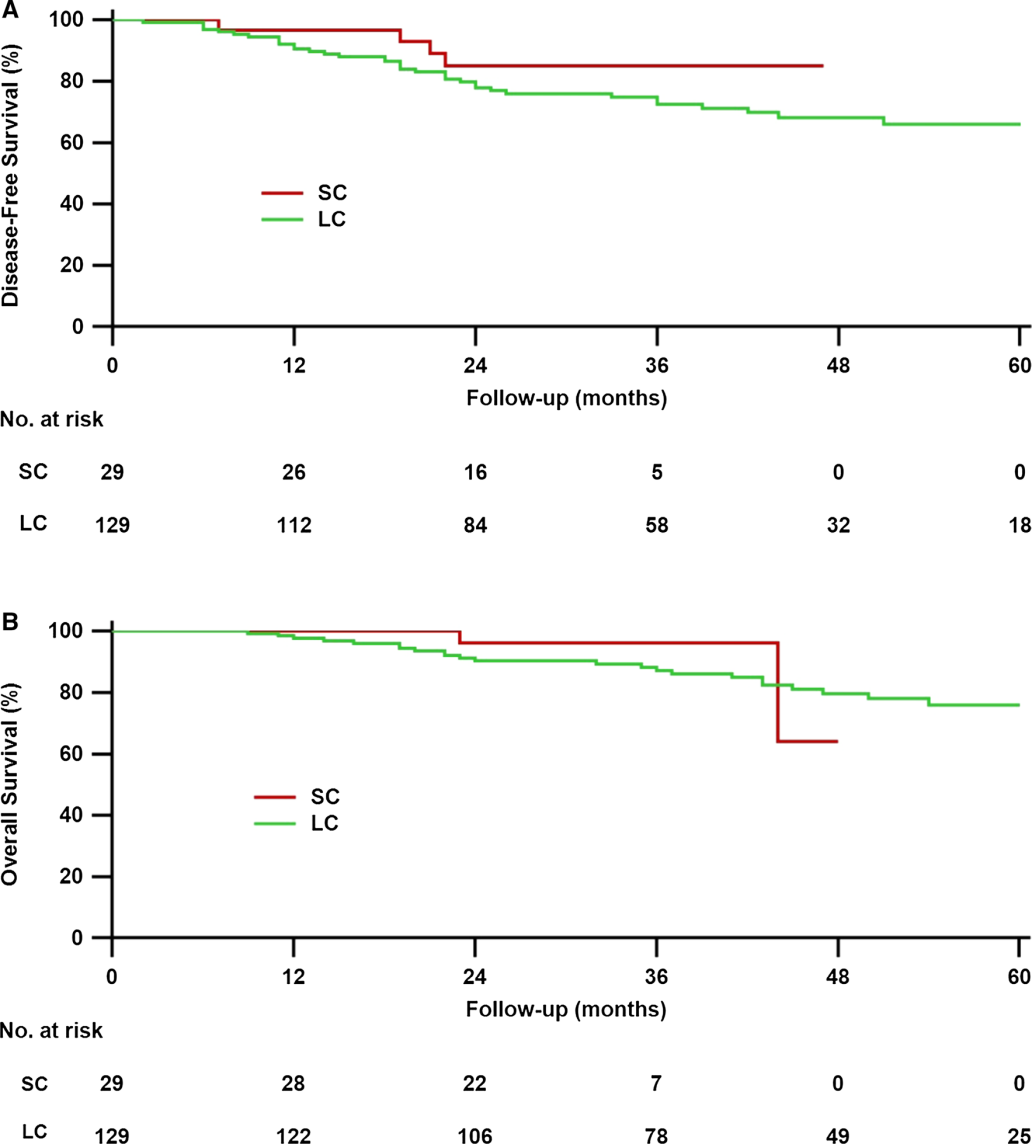
Disease-free survival (**a**) and overall survival (**b**) for different radiotherapy duration. SC = short-course radiotherapy; LC = Long-course chemoradiotherapy

On the last day of the final follow-up (August 19, 2020), 25 patients had died, and the median follow-up period for the surviving patients was 39 months. Figure 1 displays the Kaplan–Meier curve for overall survival of rectal cancer patients grouped by SC and LC. At 3 years, the DFS and OS rates in the SC and LC groups were, 84.9 vs. 72.4% (*P* = 0.273) and 96.2% versus 87.2% (*P* = 0.510), respectively. The median survival in patients receiving SC was 29.5 months, whereas the median survival in patients receiving LC was 41.5 months.

### Pathological response to therapy

As shown in Table 2, nodal downstaging was found in 93.7% of the patients treated with SC, and 72.5% of the patients treated with LC (*P* = 0.011). pCR rates in the SC group and LC group were, 25% versus 18.1% (*P* = 0.375), respectively.

**Table 2:** Pathological response with different duration of radiotherapy

Clinical variable	Radiotherapy duration		*χ*^2^	*P* value
	SC (%)	LC (%)		
*ypT*				
0 1 2 3 4	8 (25.00) 4 (12.50) 9 (28.13) 10 (31.25) 1 (3.12)	25 (18.12) 8 (5.80) 26 (18.84) 23 (16.67) 56 (40.57)	22.16	< 0.01*
*T downstaging*				
No Yes	12 (37.50) 20 (62.50)	73 (52.90) 65 (47.10)	2.46	0.12*
*ypN*				
0 1 2	24 (75.00) 8 (25.00) 0 (0.00)	103 (74.64) 23 (16.67) 12 (8.69)	–	0.15**
*N downstaging*				
No Yes	2 (6.25) 30 (93.75)	38 (27.54) 100 (72.46)	6.54	0.01*
*pCR*				
Yes	8 (25.00)	25 (18.12)	0.79	0.38*
No	24 (75.00)	113 (81.88)		

pCR, complete pathological response

*: χ^2^ test, **: Fisher exact test

## Discussion

In the current era of finding an accurate treatment for rectal cancer, insights into the effects of preoperative treatment, and determination of the best form of therapy is very important. This study showed no difference in DFS, OS, and pCR rates between the two different modes of preoperative treatments. In a randomized study by Bujko et al. [6], T3/4 stage patients receiving chemoradiation (50·4 Gy in 28 fractions of 1·8 Gy, bolus 5-fluorouracil, and leucovorin) were compared with patients who underwent radiation therapy (5 × 5 Gy), and the study reported no significant difference in the 4-year OS (66% vs. 67%, respectively) and DFS (58.4% vs. 55.6%, respectively). These findings were consistent with the results of this study.

Stockholm III trial [10] was the first to compare three different radiotherapy regimens (SC with immediate surgery, SC with delayed surgery, and LC with delayed surgery). However, the trial revealed that there was no significant difference in OS and DFS among the three groups. Besides, the pCR rate in the SC group with delayed surgery (10%) was superior to the other two groups [11]. In the present study, the pCR rate in the SC group (25%) was found to be better compared to the LC group (18.1%), and both were higher than 10%. This may be because the SC group received more preoperative chemotherapy. A previous meta-analysis found that LC presented a better pCR rate compared with the SC without chemotherapy, meaning that chemotherapy may enhance the efficacy of preoperative treatment [12]. Another randomized phase III study by Bujko et al. [13] compared patients receiving radiotherapy (5 × 5 Gy) and three cycles of FOLFOX4 with those receiving 50.4 Gy in 28 fractions combined with two 5-day cycles of bolus 5-Fu 325 mg/ m^2^/day and leucovorin 20 mg/m^2^/day during the first and fifth week of irradiation along with five infusions of oxaliplatin 50 mg/m^2^ once weekly. This study found that the pCR, DFS and OS in the two groups were, 16% versus 12% (*P* = 0.17), 53% versus 52% (*P* = 0.85), and 73% versus 65% (*P* = 0.046), respectively, thus confirming the importance of adequate chemotherapy. In the present study, we found better nodal downstaging in the SC group. However, this is not consistent with the results of the study by Brandon et al. [14], which found that the LC group were more likely to have nodal (25% vs 19%) downstaging, and pCR (15% vs 6%) compared with the SC group (all *P* < 0.05). SC itself has a similar biological effective dose as LC [15]. The large fractions used in SC can be more efficient in inducing both the innate and adaptive anti-tumor immunity, and eventually increase the biologic effects of concomitant and consolidation chemotherapy [16]. This results from the release of antigens due to the breakdown of tumoral cells, and the presentation of the antigens to T cells [17]. This difference may also be associated with the higher preoperative chemotherapy in the SC group in this study and SC with delayed surgery is also reported to have a satisfactory downstaging effect [18]. A matched pair analysis also observed that patients treated with SC and sequential FOLFOX Chemotherapy had improved rates of downstaging compared to the matched LC cohort [19]. This was precisely because early chemotherapy is likely to improve the overall therapeutic effect, hence complete neoadjuvant therapy has been proposed. There are two main proposed treatment modes: first chemoradiotherapy, and then consolidation chemotherapy, or the reverse order, induction chemotherapy first, and then chemoradiotherapy. In a multicentre, phase 2 trial by Julio et al. [20], 292 patients with stage II or III rectal cancer were divided into four groups and received zero, two, four, or six cycles of consolidation chemotherapy after preoperative chemoradiotherapy. After a median follow-up of 59 months, patients who received consolidation chemotherapy were found to have improved DFS (*P* < 0.05), and there were differences in survival between groups in patients who received at least one cycle of FOLFOX. In another phase III study [21] (STELLAR trial) in China, comparing short-course radiotherapy followed by chemotherapy with long-course chemoradiotherapy in LARC, the results showed that pCR rates in the experimental and control group were 18.6% vs. 5.4% (*P* = 0.029), respectively. These findings were consistent with the pCR rates reported in this study (18.1–25%).

The potential economic benefit cannot be ignored during the formulation of treatment. Using the micro-cost calculation method, Hanly et al. reported that SC is cheaper than LC [22]. Another study [23] analyzed the cost-effectiveness of immediate surgery after SC and LC with delayed surgery and showed that SC was the most cost-effective strategy. However, LC was also found to be a cost-effective approach for patients with distal tumors. Wang et al. [24] considered the economic benefits of both short-term and long-term radiotherapy after chemotherapy. Although the total cost of SC was much higher than that of LC ($78,937 and $38,140 respectively), the final result that was calculated through quality-adjusted life months (QALMs), found that SC was more cost-effective.

The National Comprehensive Cancer Network (NCCN) guidelines recommend SC as an acceptable alternative to LC except for patients with T4 stage rectal cancer [25]. Additionally, the American Society for Radiation Oncology (ASTRO) guidelines state that for patients with a high risk of circumferential resection margin positive or difficulty in R0 resection, LC should be used, otherwise both radiotherapy methods can be used [26]. Both SC and LC are recommended options for neoadjuvant treatment of LARC, however, the decision to choose one or the other is based on several considerations, including (1) LC is favored when a patient is at a higher risk of positive surgical margin or when tumors are distal and/or bulky and would benefit from downstaging, (2) SC followed by consolidation chemotherapy may be the most promising order for total neoadjuvant therapy (3) LC is the preferred approach when nonoperative management is being considered as it increases the chances of a complete clinical response compared with SC, (4) SC may be used in elderly and frail patients with comorbid conditions, such as heart failure since it is better tolerated than LC due to lower toxicity, and (5) SC may be used in countries with low health-care budgets or medical centers with long waiting lists because it is less expensive and more convenient.

There are still limitations to this study. Firstly, the imbalance in the number of patients receiving SC and LC may cause deviations in the results. More cases need to be included in future studies for more convincing results. Another limitation is the short follow-up time, thus evaluating long-term outcomes is uncertain. Therefore, follow-up time needs to be further extended. Then the limitations of preoperative staging should be taken into account. A meta-analysis on the diagnostic accuracy of MRI in rectal cancer patients showed that the specificities of MRI for the T category and lymph node involvement were only 75% and 71% [27]. In a future study, diffusion-weighted imaging (DWI) may be considered for improved preoperative staging, due to the high accuracy in evaluating colorectal diseases [28] and especially for poor risk patients, computed tomography (CT) [29] and virtual CT colonoscopy [30] would be a better choice because they are noninvasive and less expensive with better spatial resolution in diagnosis of colorectal lesions. Lastly, although we confirmed the importance of chemotherapy in the neoadjuvant therapy in locally advanced rectal cancer patients, due to the retrospective nature of this study, it was difficult to confirm which chemotherapy regimen or cycle number would be more beneficial. Further related research needs to be carried out in the future.

## Conclusions

Short-term radiotherapy and long-term radiotherapy are both effective and safe treatment options in patients with locally advanced rectal cancer, based on pCR, DFS, and OS.

## Data Availability

The datasets used and/or analysed during the current study are available from the corresponding author on reasonable request.

## Abbreviations

SC: Short-course radiotherapy
LC: Long-course chemoradiotherapy
LARC: Locally advanced rectal cancer
pCR: Complete pathological response
DFS: Disease-free survival
OS: Overall survival
MRI: Magnetic resonance imaging
CTV: Clinical target volume
IMRT: Intensity-modulated radiotherapy
IQR: Interquartile range
QALMs: Quality-adjusted life months
TAAs: Tumor associated antigens
CRM: Circumferential resection margin
DWI: Diffusion-weighted imaging
CT: Computed tomography
